# Mechanism of Xiezhuo Huayu Yiqi Tongluo Formula in the Treatment of Uric Acid Nephropathy Based on Network Pharmacology, Molecular Docking, and In Vivo Experiments

**DOI:** 10.1155/2023/6931644

**Published:** 2023-02-21

**Authors:** Lifei Fan, Yuqin Guo, Qingmei Wu, Tingting Hu, Xiaomei Chen, Jing Guo, Ying Liu, Yuhui Lu, Min Lin

**Affiliations:** College of Traditional Chinese Medicine, Fujian University of Traditional Chinese Medicine, Fuzhou, Fujian, China

## Abstract

**Background:**

Xiezhuo Huayu Yiqi Tongluo Formula (XHYTF) consists of 14 Chinese herbal medicines. In this study, we investigated the potential mechanism of XHYTF in the treatment of uric acid nephropathy (UAN) through network pharmacology, molecular docking, and in vivo methods.

**Methods:**

Using various pharmacological databases and analysis platforms, information on the active ingredients and targets of Chinese herbal medicine was collected, and UAN disease targets were retrieved using OMIM, Gene Cards, and NCBI. Then common target proteins were integrated. A Drug-Component-Target (D-C-T) map was constructed to screen core compounds and build a protein-protein interaction (PPI) network. Further, Gene Ontology (GO) enrichment analysis and Kyoto Encyclopedia of Genes and Genomes (KEGG) pathway analysis were performed for common targets, and a Drug-Component-Target-Pathway (D-C-T-P) network diagram was constructed. The molecular docking simulation was performed to verify the binding affinity between core components and hub targets. Subsequently, the UAN rat model was established, followed by the collection of serum and renal tissues. The expression levels of indicators in the serum were determined using an enzyme-linked immunosorbent assay. The pathological changes of renal tissues were detected using H & E staining and Masson staining. The expression of related proteins in renal tissue was detected by western blot.

**Results:**

In the study, 216 active ingredients and 439 targets in XHYTF were screened, and 868 targets were identified as being related to UAN. Among them, 115 were common targets. Based on the D-C-T network, quercetin, luteolin, *β*-sitosterol, and stigmasterol were observed to be the key active ingredients of XHYTF that were effective against UAN. The analysis of the PPI network revealed TNF, IL6, AKT1, PPARG, and IL1*β* as the 5 key targets. GO enrichment analysis revealed that the pathways were mainly concentrated in cell killing, regulation of signaling receptor activity, and other activities. Subsequently, KEGG pathway analysis revealed that multiple signaling pathways, including the HIF-1, PI3K-Akt, IL-17, and other signaling pathways, were closely related to the action of XHYTF. All 5 key targets were confirmed to interact with all core active ingredients. In vivo experiments indicated that XHYTF significantly reduced blood uric acid and creatinine levels, alleviated inflammatory cell infiltration in kidney tissues, reduced the levels of serum inflammatory factors such as TNF-*α* and IL1*β*, and ameliorated renal fibrosis in rats with UAN. Finally, western blot revealed decreased levels of PI3K and AKT1 proteins in the kidney, which confirmed the hypothesis.

**Conclusion:**

Collectively, our observations demonstrated that XHYTF significantly protects kidney function, including alleviation of inflammation and renal fibrosis via multiple pathways. This study provided novel insights into the treatment of UAN using traditional Chinese medicines.

## 1. Introduction

Uric acid nephropathy (UAN) is caused by disrupted purine metabolism, resulting in excessive blood uric acid levels, excessive or reduced renal excretion, and supersaturated deposition of uric acid in the serum, resulting in deposits in the medulla, interstitium, or distal collecting duct, causing kidney damage. Patients with hyperuricemia had a 2.14-fold higher risk of developing chronic kidney disease (CKD) than patients with normal UA levels [1]. As observed in animal models of UAN and by studying the changes in human renal pathology, the main pathological manifestations of UAN were observed to be diffuse tubulointerstitial lesions, renal arteriosclerosis, and tubular epithelial cell injury [2–4]. At present, the treatment of UAN mainly focuses on inhibiting uric acid production and promoting uric acid excretion using first-line drugs such as allopurinol and benzbromarone. However, their clinical application is often limited because of their serious side effects. Therefore, it is here that various researchers' different studies converge to explore the intervention measures of UAN, which are crucial.

Traditional Chinese medicine (TCM) has its own unique understanding of the pathogenesis of UAN. Numerous studies indicated that TCM may be beneficial in treating UAN in a multitarget and multipathway manner [5–7]. Xiezhuo Huayu Yiqi Tongluo Formula (XHYTF) is the clinical experience of our research group, which is clipping from SiMiao Wan of Cheng Fang Bian Du (also named as Into Easy To Read, 1904 A. D.) written by Dr. Bing-cheng Zhang. It consists of 14 types of TCMs, namely, Coicis Semen (Yiyiren in Chinese, YYR), Smilacis Glabrae Rhixoma (Tufuling in Chinese, TTL), Persicae Semen (Taoren in Chinese, TR), Pseudobulbus Cremastrae Seu Pleiones (Sancigu in Chinese, SCG), Spatholobus Suberectus Dunn (Jixueteng in Chinese, JXT), Hedysarum Multijugum Maxim (Huangqi, HQ), Phellodendri Chinrnsis Cortex (Huangbo in Chinese, HB), Pheretima (Dilong in Chinese, DL), Codonopsis Radix (Dangshen in Chinese, DANGS), Radix Salviae (Danshen in Chinese, DANS), Radix Rhei Et Rhizome (Dahuang in Chinese, DH), Cyathulae Radix (Chuanniuxi in Chinese, CNX), Plantaginis Semen (Cheqianzi, in Chinese, CQZ), and Rhizoma Atractylodis (Cangzhu in Chinese, CZ). During the past decades, some extracts of traditional Chinese medicine in this formula have been proven to play an important role in the treatment of uric acid nephropathy. For example, studies have shown that the root extract of *Rhizoma smilacis glabrae* has antioxidant, anti-inflammatory, and immune regulation properties, lowering uric acid levels, reducing inflammatory cell infiltration of kidney tissue, protecting kidneys, and so on [8]. Rhein, the active ingredient of *Chinese rhubarb*, can inhibit inflammatory damage and has a protective effect on UAN [9]. The therapeutic mechanism of XHYTF on UAN may involve a complex system of multiple components, multiple targets, and multiple pathways, which requires a more extensive study in accordance with the overall strategy.

**Figure 1:**
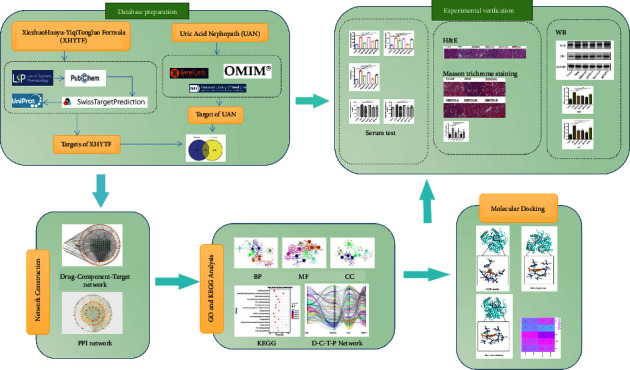
The overall flowchart of this study.

Network pharmacology is an emerging research approach, which acts as a catalyst for revealing the biological basis of herbal formulations [10]. By integrating multidisciplinary information, a component-protein/gene-pathway network model can be obtained to systematically analyze the potential mechanisms of drugs, which can be used to understand the mechanism of action of TCM in the treatment of diseases. In this study, based on the network pharmacology research strategy, we assessed the interaction of XHYTF with the correlative molecular network of UAN through target prediction, network construction, pathway enrichment analysis, molecular docking, and other biological information technologies combined with animal experiments. The predicted results were verified to comprehensively explore the mechanism of action of XHYTF in UAN. The detailed flow chart of the study design is given in Figure 1.

## 2. Materials and Methods

### 2.1. Network Pharmacology

#### 2.1.1. Target Fishing of the Active Ingredients and Construction of Composite Network

The active ingredients and targets of XHYTF were mainly mined from the online Traditional Chinese Medicine Systems Pharmacology (TCMSP) database (https://lsp.nwsuaf.edu.cn/tcmsp.php). The threshold was set to oral bioavailability (OB) ≥30% and drug likeness (DL) ≥0.18 for further filtering to obtain the active ingredients; this was supplemented by a literature search to continue including the active ingredients that had been deleted because of OB and DL thresholds but had research significance in the follow-up study. Pheretima could not be retrieved in TCMSP; therefore, the active ingredients could be collected by searching the CNKI database (https://www.cnki.net/) and using the PubChem database (https://Pubchem.ncbi.nlm.nih.gov/). SMILE structures of potential active ingredients were collected and imported into the SwissTargetPrediction database (https://www.swisstargetprediction.ch/) to predict potential targets. Selection probability >0 and TOP10 target genes were used as targets of the Pheretima. All targets were normalized to gene ID using the annotation file of the UniProt database (https://www.uniprot.org/). The screened active ingredients and potential targets of XHYTF were imported into Cytoscape 3.7.1 (Institute of Systems Biology, US) to construct a network diagram of Drug-Component-Target (D-C-T).

#### 2.1.2. Database Building of Disease Targets

The known targets related to UAN were obtained using the key word “urine acid nephropathy” from the GeneCards database (https://www.genecards.org/), the Online Mendelian Inheritance in Man (OMIM, https://omim.Org/), and the NCBI database (https://www.ncbi.nlm.nih.gov/gene). The screening condition of the GeneCards database was “Relevance Score” >10. The online tool Venny 2.1 (https://bioinfogp.cnb.csic.es/tools/venny/index.html) was used to screen the common targets of XHYTF against UAN.

#### 2.1.3. Construction of a D-C-T Network

Based on the above analyses, the compounds corresponding to common targets were matched with the drugs corresponding to the compounds. Further, Cytoscape 3.7.1 was used to construct a D-C-T network diagram, which was used for fishing the key active ingredients.

#### 2.1.4. Construction of the Protein-Protein Interaction (PPI) Network

The obtained intersection targets were uploaded to the STRING database (https://string-db.org/). The targets had the gene type *Homo sapiens* and selective PPI networks with a medium confidence level ≥0.400, and the free nodes were hidden. The PPI network information was imported into Cytoscape 3.7.1, using “Network Analyzer” to calculate the topology parameters of the protein interaction network. The node size and color reflect degree value, and the edge thickness reflects the combined score. Finally, the protein interaction network was obtained and visualized. The targets with degree values in the top 5 were selected as the core targets.

#### 2.1.5. Gene Ontology (GO) and Kyoto Encyclopedia of Genes and Genomes (KEGG) Pathway Enrichment Analyses

Gene ontology (GO) functional annotation was conducted using the ClueGO plug of Cytoscape3.7.1, and the modules Biological Process (BP), Cell Compound (CC), and Molecular Function (MF) were selected. The ClueGO network was created using Kappa statistics. Representative GO terms and pathways were selected using predefined filters. The importance of these pathways and their similarity in related genes is automatically mapped onto the network and illustrated in different interchangeable visual styles [11]. Simultaneously, the common targets of XHYTF and UAN were added to Metascape (https://metascape.org/gp/index.html#/main/Step1) to perform KEGG pathway analysis. The results with min overlap = 3, min enrichment = 1.5, and *P* < 0.01, were screened for the analysis of enrichment and sorted as per ascending *P* values. The top 15 pathways ranked by KEGG were obtained. Additionally, the OmicShare Tools online platform (https://www.omicshare.com/tools/) was used to map the bubble chart of the KEGG enrichment analysis.

#### 2.1.6. Construction of a Drug-Component-Target-Pathway Network

The 15 pathways obtained as mentioned in Section 2.1.5, their targets, corresponding compounds of the targets, and corresponding drugs of the compounds were collected to construct the network diagram of Drug-Component-Target-Pathway (D-C-T-P). The nodes in the figure represent drugs, components, targets, and pathways. The bands represent the correlation between them, and the width of the bands reflects the strength of the correlation.

#### 2.1.7. Molecular Docking

2D structures of the key active ingredients of XHYTF were downloaded from the PubChem database, and crystal structures corresponding to core targets were obtained from the RCSBPDB platform (https://www1.rcsb.org/). Autodock Vina molecular docking was conducted on the SailVina platform. Molecular docking was performed by preparing the receptor, using Biopython functional correction, preparing the ligand, preparing the docking site, etc. Finally, docking scores were extracted. The binding activity was evaluated from the docking score value, and the molecular docking results were obtained. Further, Open Babel software (Free Software Foundation, Inc., MA, USA) was used to convert “PDBQT” format to “PDB” format, and the results were visualized using PyMOL software (Schrodinger, the US). Simultaneously, the binding sites were determined on the PLIP website (https://projects.biotec.tu-dresden.de/plip-web/plip/index); the PyMOL results file format was downloaded; and binding site residues were tagged and visually processed using PyMOL software.

### 2.2. Experimental Evidence

#### 2.2.1. Materials and Chemicals

Paraformaldehyde (batch No. G1101), hematoxylin-eosin (HE, batch No. G1003), etc. were purchased from Servicebio Biotechnology Co., Ltd. (Wuhan, China). Ethyl carbamate (batch No. 20210521), absolute ethanol (batch No. 100092683), xylene (batch No. 10023418), and neutral gum (batch No. 10004160) were purchased from Sinopharm Chemical Reagent Co., Ltd. (China). Allopurinol (batch No. X20N10Y103527), ethambutol (batch No. R16S10T97786), and adenine (batch No. J10G151411) were purchased from Yuanye Biotechnology Co., Ltd. (Shanghai, China). Carboxymethyl cellulose (batch No. C12339554) was purchased from Macklin Biotechnology Co., Ltd. (Shanghai, China). Enzyme-linked immunosorbent assay (ELISA) kits for serum TNF-*α* (batch No. RJ16622) and IL-1*β* (batch No. RJ15465) were purchased from Renjie Biotechnology Co., Ltd. (Shanghai, China). RIPA lysate (batch No. AR0102), protease inhibitor (batch No. AR1178), polyvinylidene difluoride (PVDF) membranes (batch No. AR0136-02), bicinchoninic acid (BCA) protein assay kit (batch No. AR0198), SDS-PAGE kit (batch No. AR0138), HRP Goat Anti-Rabbit IgG (H + L) (batch No. SA00001-2), HRP Goat Anti-Mouse IgG (H + L) (batch No. SA00001-1), 5× protein buffer (batch No. AR1112), chemiluminescence liquid (batch No. AR1111), and electrophoresis buffer solution (batch No. AR0139) were purchased from BOSTER Biological Technology Co., Ltd. (Wuhan, China). Rapid membrane transfer fluid (batch No. SS0405A) was purchased from Shuohua Biotechnology Co., Ltd. (Zhejiang, China). Antibodies against PI3K (batch No. 20584-1-AP), Akt (batch No. 60203-2-Ig), and GAPDH (batch No. 60004-1-Ig) were purchased from Proteintech Group, Inc. (Chicago, USA).

Adenine + Ethambutol mixed suspension: first, 500 mg sodium carboxymethylcellulose was dissolved in 100 mL distilled water and configured into 5% sodium carboxymethyl cellulose solution for reserve. The suspensions of adenine (100 mg/kg·d) and ethambutol (250 mg/kg·d) were prepared by suspending 100 mg adenine and 250 mg ethambutol in 5% carboxymethylcellulose sodium solution, stored at −20°C, and brought to room temperature before use. Allopurinol solution was prepared by mixing 50 mg with normal saline to obtain 100 mL solution (for use and storage, procedures followed were the same as those mentioned above).

#### 2.2.2. Source and Preparation of XHYTF

The components of XHYTF used in the study were Coicis Semen 30 g, Smilacis Glabrae Rhizoma 30 g, Persicae Semen 10 g, Pseudobulbus Cremastrae seu Pleiones 9 g, Spatholobus suberectus Dunn 20 g, Hedysarum multijugum Maxim 30 g, Phellodendri chinensis cortex 12 g, Pheretima 15 g, Codonopsis Radix 15 g, Radix Salviae 20 g, Radix Rhei Et Rhizome 3 g, Cyathulae Radix 15 g, Plantaginis Semen 15 g, and Rhizoma Atractylodis 10 g (The Third People's Hospital of Fujian Province). To them, 10 times the volume of water was added, followed by soaking for 30 min. The solutions were boiled twice, 1 h each time. Further, concentrations of 4.2 g/mL (high dose solubility) were obtained using a rotary evaporator. The samples were stored in a refrigerator at −20°C, brought to room temperature before use, and diluted as required.

#### 2.2.3. Animals and Models

A total of 30 male Sprague-Dawley (SD) rats were purchased from the Hangzhou Medical College (Certificate No.: SCXK (Zhe) 2019-0002, Hangzhou, China). The animals were reared in the SPF feeding room of the Fujian University of Traditional Chinese Medicine Experimental Animal Center (FJTCM IACUC-2021145). The animals were group-housed with a 12 h light/12 h dark cycle, and the temperature was set at 22–25°C. The rats were randomly divided into 6 groups: control, model, allopurinol, XHYTF low-dose (XHYTF-L), XHYTF medium-dose (XHYTF-M), and XHYTF high-dose (XHYTF-H) groups. The rats were allowed to adapt to the environment for 1 week before experiments. Weighing and recording were done before each experiment. The dosage was calculated based on the adult body weight of 70 kg. Hence, the dosage of XHYTF in rats was calculated according to body surface area [12]. Once a day in the morning for 21 consecutive days, rats in the control group were subjected to normal saline intragastric administration, and those in the other groups were administered with adenine (100 mg/kg·d) and ethambutol (250 mg/kg·d) suspension to establish the UAN model. The control and model groups were intragastrically administered with equal volumes of normal saline; the allopurinol group was intragastrically administered with 5 mg/kg allopurinol. The XHYTF-H, XHYTF-M, and XHYTF-L groups were intragastrically administered with 42.12, 21.06, and 10.53 g/kg XHYTF, respectively, once a day in the afternoon for 21 consecutive days.

After the last administration, the rats were fasted for 12 h and deprived from water of 4 h. They were anesthetized by intraperitoneal injection with ethyl carbamate solution. Blood was collected through the abdominal aorta, and serum samples were obtained by centrifugation at 3500 rpm for 10 min, which were stored at −80°C. The bilateral kidneys of rats in each group were separated and weighed. The right kidney tissue was quickly and carefully separated on an ice plate, and a part of the right kidney tissue was cut, frozen in liquid nitrogen, and stored at −80°C for western blot analysis. Furthermore, partial kidney tissue was cut and fixed with 4% buffered paraformaldehyde for morphological analysis. All the procedures were approved by the Experimental Animal Welfare Committee of the Fujian University of Traditional Chinese Medicine.

#### 2.2.4. Serum Test

The levels of serum uric acid (SUA), serum creatinine (Scr), and blood urea nitrogen (BUN) in rats in each group were detected using an automatic biochemical analyzer. The levels of TNF-*α* and IL-1*β* were detected using ELISA according to the manufacturer's protocol.

#### 2.2.5. Pathological Analysis of Renal Tissue

Renal tissue was dehydrated and transparent with gradient ethanol and xylene, embedded in paraffin, and stained with HE and Masson's trichrome staining. The sections were sealed with neutral gum. The renal tissue sections were carefully observed under a microscope, and images were taken. Three visual fields in each section were randomly selected, the proportion of collagen-positive area (blue) in the kidney was measured, and the target area of tissue was selected for 200-fold imaging. ImageJ analysis software was used to measure the collagen pixel area of Masson-stained sections, and the proportion of collagen deposition area was used to evaluate interstitial fibrosis.

#### 2.2.6. Western Blot

Renal tissues of each group were lysed with lysis buffer, and the supernatant was collected after centrifugation. A BCA protein concentration assay kit was used to determine the protein concentration. The same amount of protein was separated by SDS-PAGE and transferred to a PVDF membrane, and the expression levels of target proteins were detected. Protein expression levels were normalized to GAPDH expression levels.

#### 2.2.7. Statistical Analyses

Relevant data were analyzed using SPSS 26.0 statistical software (SPSS, Chicago, USA). All results were presented as mean ± standard deviation (SD), and a univariate ANOVA was used to test the significance of differences. When variance was not uniform, the rank sum test was used. For the nonparametric tests, the Kruskal–Wallis test was used for the analysis. *P* < 0.05 was considered significant.

## 3. Results

### 3.1. Active Ingredients and Target Genes of XHYTF

A total of 216 compounds were screened from 14 herbs of XHYTF with corresponding gene proteins, of which 6, 15, 18, 3, 23, 18, 25, 34, 17, 60, 10, 4, 7, and 4 compounds were obtained from Coicis Semen, Smilacis, Pseudobulbus Cremastrae Seu Pleiones, Spatholobus Suberectus Dunn, Hedysarum Multijugum Maxim, Phellodendri Chinrnsis Cortex, Pheretima, Codonopsis Radix, Radix Salviae, Radix Rhei Et Rhizome, Cyathulae Radix, Plantaginis Semen, and Rhizoma Atractylodis, respectively (Table 1). Among them, 15 compounds were common to various herbs and were numbered *A*1, *A*2, *A*3, *B*1, *C*1, etc. After deleting repeated targets, 439 corresponding target genes were obtained from these active ingredients.

### 3.2. UAN-Related Targets and D-C-T Network

**Figure 2:**
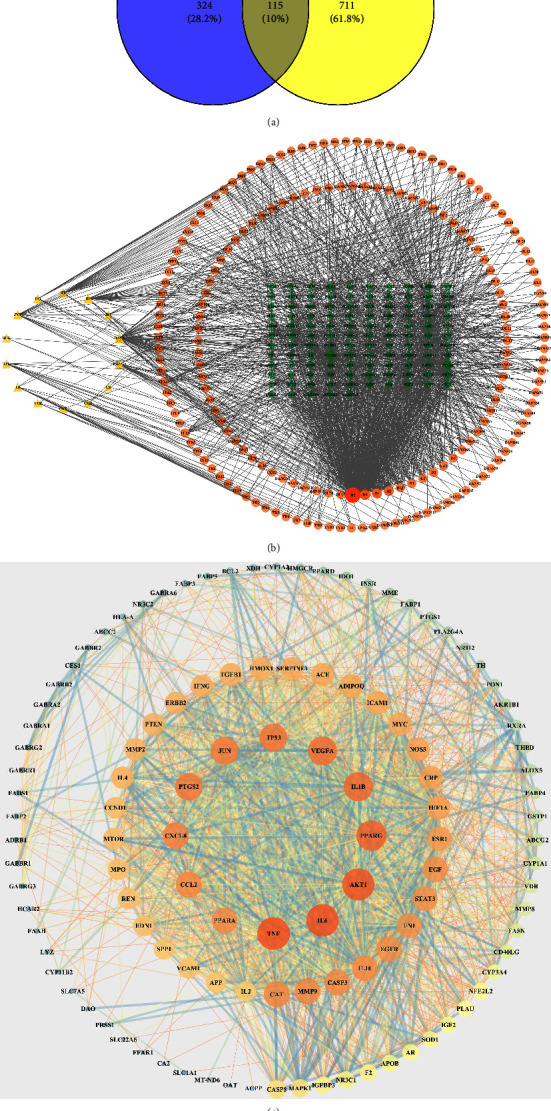
Target network analysis. (a) Venn diagram of the drug targets and disease proteins. (b) D-C-T network interactions of the 115 predicted targets. Yellow triangles represented drugs, orange circles represented compounds, and green diamonds represented targets. (c) PPI interaction network.

Overall, 358,428 and 36 UAN-related targets were respectively identified through the GeneCards, OMIM, and NCBI databases. A total of 822 disease targets were obtained after all targets were integrated and repeated values were deleted. The Venny 2.1 online tool was used to draw a Venn diagram for drug targets and disease targets (Figure 2(a)), and 115 intersection target genes of XHYTF and UAN were obtained. Cytoscape 3.7.1 was used to construct a “D-C-T” network (Figure 2(b)), which contained 318 nodes and 1597 edges. Yellow triangles represented drugs, orange circles represented compounds, and green diamonds represented targets. Notably, the size of the node represents the degree value of the node. The darker the color and larger the area, the more edges sent out by the node. The closer the correlation degree of D-C-T, the more likely it is to play a crucial role. According to network parameter analysis, the top 4 components by degree value were quercetin (*B*2), luteolin (*D*4), beta-sitosterol (*B*1), and stigmasterol (*A*2).

### 3.3. PPI Network Analysis

A PPI network diagram was drawn according to degree and combine score values, with node area representing degree value and line thickness representing combine score value. The PPI network consisted of 115 nodes with 1823 edges representing PPIs (Figure 2(c)). The average number of neighbors of the PPT network was 31.704, and the clustering coefficient was 0.660. Moreover, the larger the node, the brighter the color, indicating a larger grade value. The importance of the target is determined by the degree value. Finally, TNF (79), IL6 (78), AKT1 (77), PPARA (72), and IL1B (69) were defined as the key targets by degree sequencing.

### 3.4. GO and KEGG Pathway Enrichment Analyses

**Figure 3:**
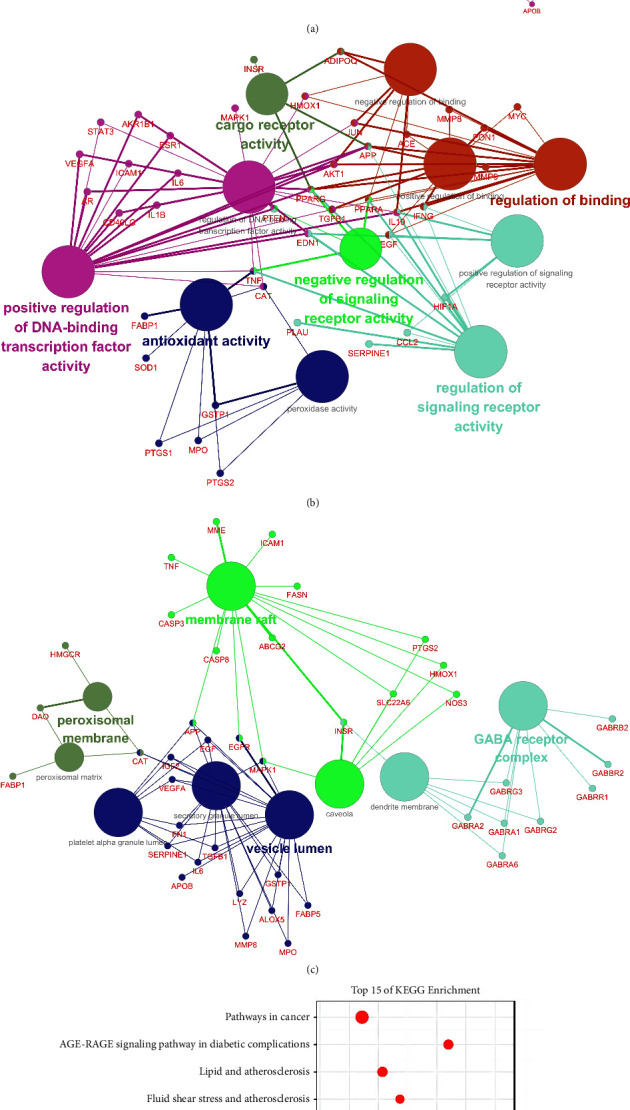
Enrichment analysis and gene-pathway network analysis. (a) GO-biological process enrichment network. (b) GO-molecular function enrichment network. (c) GO-cell component enrichment network. (d) KEGG pathway enrichment of the common targets of XHYTF on UAN.

Based on ClueGO plugins of Cytoscape3.7.1 software, 115 common targets were annotated and analyzed in BP, MF, and CC for GO classification and functional annotation, and biological processes with *P* < 0.05 were screened. The GO item with a smaller *P* value was more closely related to the XHYTF treatment UAN. GO analysis results revealed that 824 items were obtained from BP analysis. The results were reclustered, and “Min Level: 0 and Max Level: 2” was selected for visualization. It mainly involved cell killing, regulation of growth, rhythmic processes, regulation of plasma lipoprotein particle levels, etc. (Figure 3(a)). MF analysis yielded 80 entries, which mainly included regulation signaling receptor activity, antioxidant activity, and positive regulation of DNA-binding transcription factor. “Min Level: 0 and Max Level: 2” were selected for visualization (Figure 3(b)). Overall, 9 items were obtained from CC analysis, mainly involving the GABA receptor complex, vesicle lumen, secretory granule lumen, platelet alpha granule lumen, etc. (Figure 3(c)). Each node represents a term. The line reflects the correlation between terms. The color of the node reflects its enrichment classification, and the size of the node indicates its enrichment degree.

In total, 184 KEGG pathways were screened from the Metascape database. The analysis revealed that it was mainly enriched in the AGE-RAGE signaling pathway in diabetic complications, the HIF-1 signaling pathway, the PI3K-Akt signaling pathway, and the TNF signaling pathway. The top 15 pathways were visualized in the form of a bubble chart according to the *P* values (Figure 3(d)).

### 3.5. D-C-T-P Network

**Figure 4:**
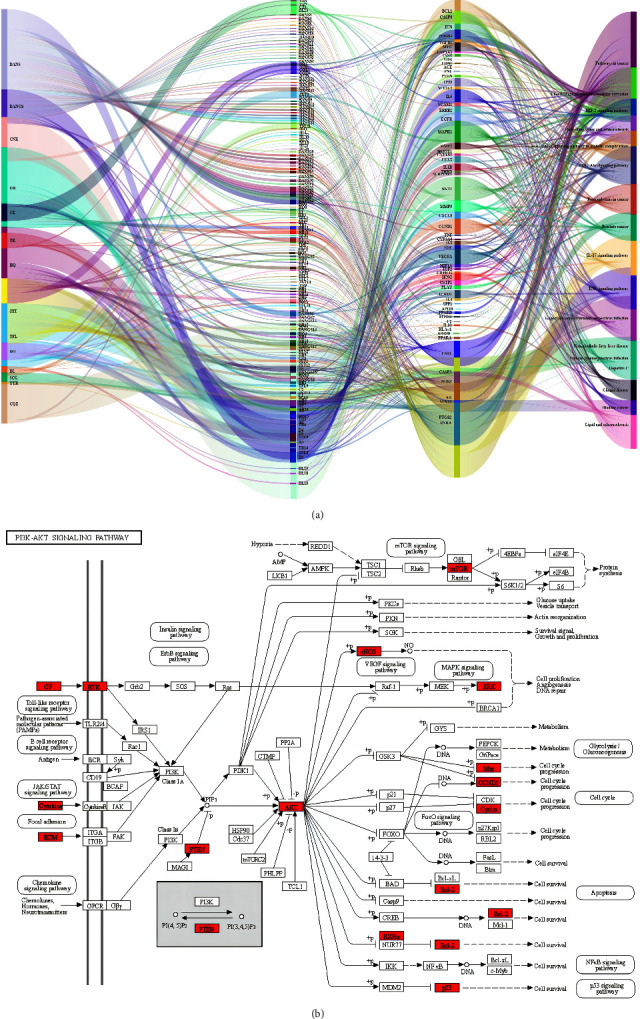
(a) D-C-T-P network interactions of the 115 predicted targets. (b) Role of XHYTF in the target pathways of UAN. The red rectangles represent the crucial target genes that mediate important downstream signals in the pathway.

Based on the top 15 KEGG enrichment results, the mechanism of action of XHYTF against UAN was comprehensively described by plotting the D-C-T-P Sankey diagram (Figure 4). The diagram included 14 herbs and 159 compounds, which act on 15 pathways such as IL-17, TNF, and PI3K-Akt signaling pathways through 60 targets.

### 3.6. Molecular Docking Verification

**Figure 5:**
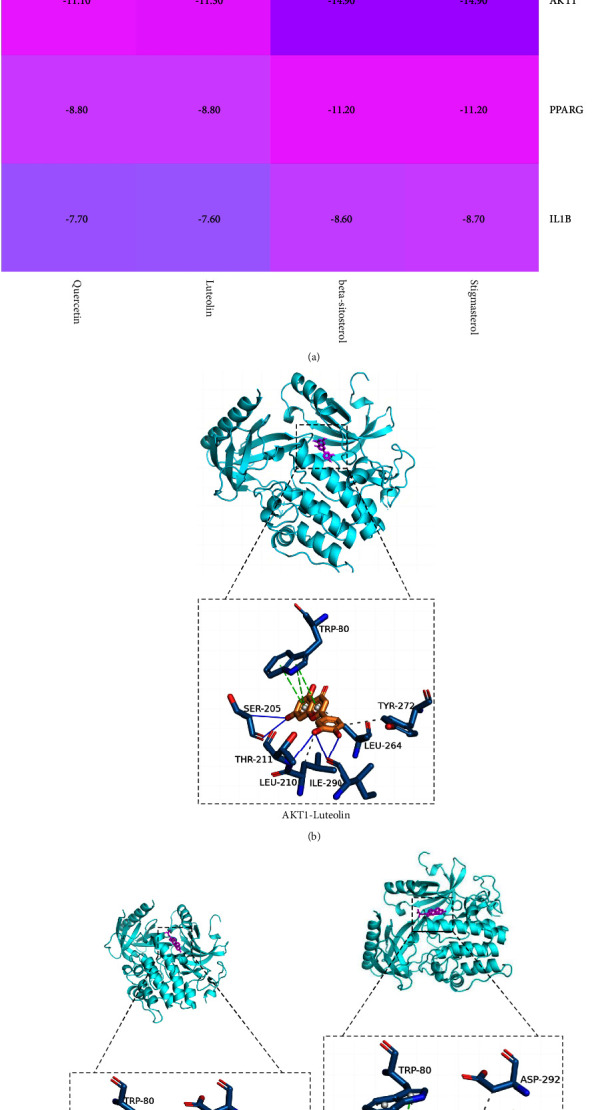
Molecular docking of core targets and active components. (a) Heat maps show docking scores of core targets combining with core components of XHYTF; (b) AKT1-luteolin; (c) AKT1-beta-sitosterol; (d) AKT1-stigmasterol.

The first four key components obtained from D-C-T network analysis were docked with the five potential core targets obtained from PPI network analysis. The docking score represents the affinity between the protein receptor and the docking ligand. The lower the docking score, the better the affinity and the more stable the binding of the component to the target (Figure 5(a)). The docking fraction between 20 pairs of components and targets was lower than −5 (kcal/mol), indicating that better binding affinity existed between 4 compounds and 5 targets. This confirmed the scientific efficacy of XHYTF in treating UAN. Among them, the binding energies of the core components, namely, stigmasterol and *β*-sitosterol, and the core target, namely, AKT1, were −14.9 kcal·mol^−1^, which were the smallest among the 20 docking results. These results indicated that stigmasterol and *β*-sitosterol may exhibit therapeutic effects in UAN by acting on AKT1. We visualized the binding posture and binding site residues of the interaction between the three pairs of composite targets with the lowest docking scores (Figures 5(b)–5(d)).

### 3.7. Evaluation and Analysis of UAN by XHYTF In Vivo

#### 3.7.1. Serological Observation

**Figure 6:**
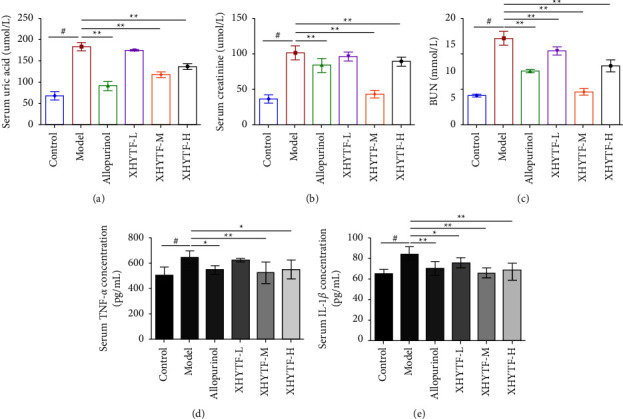
XHYTF improves kidney function and reduce the release of proinflammatory cytokines in UAN rats. XHYTF reduced serum uric acid (a), serum creatinine (b), BUN (c), serum TNF-*α* concentration (d), and serum IL-1*β* concentration (e). ^#^*P* < 0.01, vs. control; ^*∗*^*P* < 0.05, ^*∗∗*^*P* < 0.01, vs. model.

The levels of SUA, Scr, and BUN were detected. Compared with the control group, the levels of SUA, Scr, and BUN in the serum of rats in the model group were significantly increased (*P* < 0.01), indicating the successful establishment of the UAN rat model. Compared with the model group, SUA, Scr, and BUN levels were significantly decreased in the allopurinol and all XHYTF groups (*P* < 0.05 or *P* < 0.01) (Figures 6(a)–6(c)). These results suggested that XHYTF can reduce uric acid and improve renal insufficiency in rats with UAN.

To study the relationship between inflammation and UAN, serum TNF-*α* and IL-1*β* levels in rats were detected using ELISA. Compared with the control group, the expressions of proinflammatory factors TNF-*α* and IL-1*β* in the model group were high (*P* < 0.01), indicating that inflammation was activated in UAN. Compared with the model group, TNF-*α* and IL-1*β* levels in the allopurinol and all XHYTF groups were decreased (*P* < 0.05 or *P* < 0.01), and the effect was more significant in the XHYTF-M group (Figures 6(d) and 6(e)).

#### 3.7.2. Renal Histological Observation

**Figure 7:**
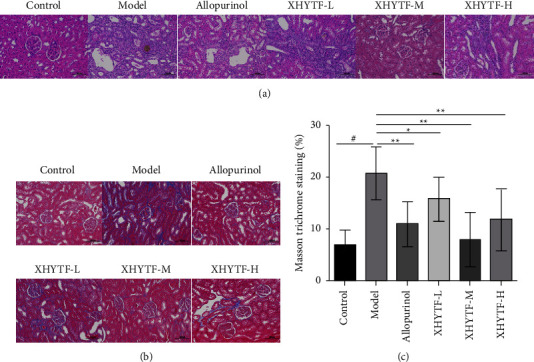
XHYTF improved the pathological changes in the kidney. (a) Micrographs of hematoxylin and eosin staining of kidneys (original magnification, 200×). (b) Micrographs of Masson's trichrome staining of kidneys (original magnification, 200×). (c) The graph showed the percentage of collagen positive area (blue) relative to the whole area. ^#^*P* < 0.01, vs. control; ^*∗*^*P* < 0.05, ^*∗∗*^*P* < 0.01, vs. model.

To evaluate the effect of XHYTF on renal histology, the bilateral kidneys of rats in each group were isolated. HE staining was performed on the right kidney of each group to observe renal pathological changes, and Masson's trichrome staining was performed to evaluate extracellular matrix protein deposition in the kidneys. In the control group (*P* < 0.01), the renal tissue structure was normal without inflammatory infiltration and uric acid crystals, and no obvious connective tissue hyperplasia was observed in the interstitium. However, renal interstitium was widely observed in the model group, with renal tubular epithelial atrophy, small size or loss of structure, lymphocyte infiltration, increased collagen fibers in the interstitium, mild interstitial fibrosis, uric acid crystals, and multinucleated giant cell phagocytosis. Compared with the model group (*P* < 0.05 or *P* < 0.01), the allopurinol and all XHYTF groups exhibited reduced renal tubular injury, uric acid crystal deposition, inflammatory cell infiltration, and interstitial fibrosis levels to varying degrees (Figure 7).

Interstitial fibrosis is a common way of renal injury in UAN. We detected extracellular matrix protein deposition in the kidney using Masson trichromatic staining. The results revealed that the proportion of collagen-positive renal tubule interstitial area increased in the model group and decreased in all other XHYTF groups.

#### 3.7.3. Verification of Expression of Proteins Related to the XHYTF-Regulated Pathways

**Figure 8:**
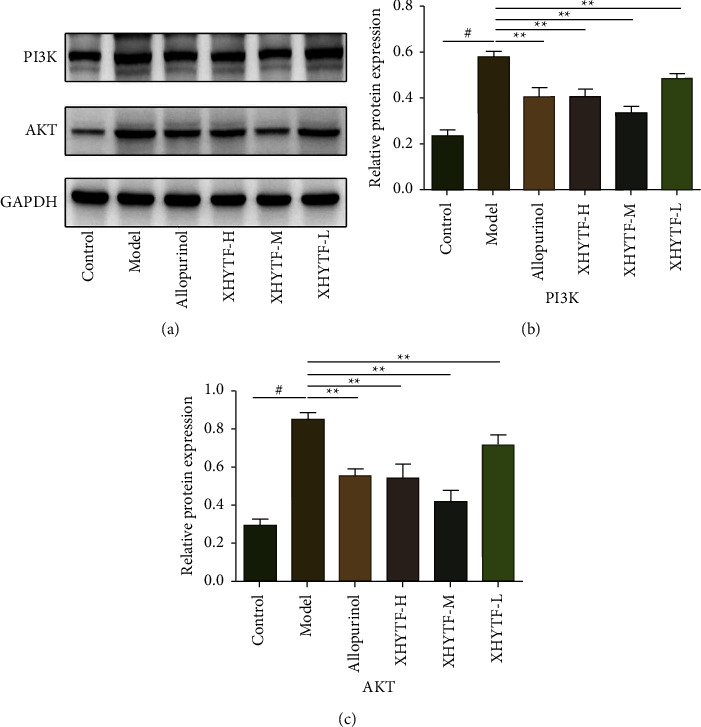
The expression of key proteins in the PI3K-Akt signaling pathway. (a) Western blotting was conducted to evaluate the protein level of PI3K, AKT, and GAPDH in the kidney tissue lysates. (b, c) The PI3K and AKT protein levels normalized by GAPDH.

To investigate whether XHYTF has a regulatory effect on the PI3K-Akt signaling pathway, western blotting was performed to detect the levels of proteins from the PI3K and Akt pathways according to the prediction results of network pharmacology and molecular docking. Compared with the control group, the protein expression of PI3K and AKT was significantly increased in the model group (*P* < 0.01), and it was significantly restored by XHYTF treatment (*P* < 0.05 or *P* < 0.01) (Figure 8).

## 4. Discussion

UAN is characterized by uric acid kidney stones, chronic interstitial nephritis, and interstitial fibrosis, which may eventually lead to chronic renal failure. The potential role of uric acid in the progression of kidney disease has been demonstrated. Epidemiological studies have consistently reported that SUA is a risk factor for CKD [13]. Elevated SUA levels are associated with tubular injury, inflammatory cell infiltration, and subsequent tubular interstitial fibrosis [14, 15]. Western therapies have focused on benzbromarone and other drugs to promote uric acid excretion by mediating reabsorption and xanthine oxidase inhibitors such as allopurinol and febuxostat to inhibit uric acid production. However, these drugs have limitations because of their low selectivity and toxicity [16–18]. TCMs have unique advantages in the treatment of UAN because of the combined characteristics of their multiple components, multiple targets, and multiple pathways. However, their complex pharmacological mechanism is difficult to explain. Network pharmacology is used to study the pathway model of “component-protein/gene-disease” and describe the complexity among biological systems, drugs, and diseases from the perspective of the network model, which is consistent with the overall philosophy of TCM [19]. XHYTF is an empirical prescription used by our research group in the clinical treatment of UAN, and its curative effect is significant. To the best of our knowledge, this is the first study to use the network pharmacology method and molecular docking simulation to analyze the network molecular mechanism of XHYTF and perform relevant verification through in vivo experiments to further explore the potential mechanism of XHYTF in UAN treatment.

The results of network pharmacology and molecular docking revealed the multitarget and multipathway characteristics of XHYTF in treating UAN. Through data mining, 216 potential bioactive ingredients and 115 UAN-related targets were identified for XHYTF. The “D-C-T” network identified quercetin, *β*-sitosterol, stigmasterol, and luteolin as the key potential active ingredients. Furthermore, through PPI network analysis of UAN targets, we obtained 5 targets with the highest degree, including TNF, IL6, AKT1, PPARA, and IL-1*β*. GO functional enrichment and KEGG pathway analyses were performed to explore the biological mechanism of XHYTF. GO analysis suggested that the targets of XHYTF in treating UAN may be involved in cell killing, regulation of growth, rhythmic processes, regulation of signaling receptor activity, the GABA receptor complex, and other processes. KEGG enrichment analysis revealed that the targets were mainly enriched in the AGE-RAGE signaling pathway in diabetic complications, lipids, and atherosclerosis, the HIF-1 signaling pathway, the PI3K-Akt signaling pathway, the TNF signaling pathway, the IL-17 signaling pathway, etc. Among the predicted key compounds, quercetin was the most promising target. It has been reported that quercetin can inhibit renal inflammation and fibrosis in UUO and LPS mouse models by decreasing expressions of IL-18, IL-1*β*, TNF-*α*, and fibrosis area in Masson staining and inhibiting collagen expression [20]. In addition, quercetin has a protective effect on renal tubular epithelial cell injury induced by oxidative stress in hyperoxaluria rats [21]. Therefore, we verified the binding ability of the key compounds to the five core genes through molecular docking simulation, and the docking results revealed that these compounds could stably bind to the active pocket of the core protein. Moreover, in vivo experimental results confirmed that XHYTF may reduce renal fibrosis and inflammatory infiltration in rats with UAN, release of proinflammatory cytokines (TNF-*α* and IL-1*β*) and uric acid levels, and play a role in improving renal function through the targeted regulation of the PI3k/Akt pathway, which verified the accuracy of our analysis.

Network pharmacology suggested that some of the anti-UAN targets of XHYTF were involved in the above pathways, and molecular docking results revealed that XHYTF exhibited a high affinity for binding with the important targets in the above pathways. Collectively, we speculated that XHYTF may play an antifibrosis and anti-inflammatory role in UAN through targeted regulation of the PI3K/AKT pathway. Among the pathological features of UAN, inflammatory reaction and fibrinosis are the fundamental pathological mechanisms, whereas the SUA-induced inflammatory mechanism pathway has been proven to be the main pathophysiological basis for the development of renal tubular injury and interstitial fibrosis and has been well-accepted as playing an important role in UAN injury [22]. Uric acid can trigger an inflammatory cascade by inducing the production of proinflammatory cytokines such as TNF-*α* and IL-1*β*, which can cause local renal tubular injury and renal tubular interstitial fibrosis [23, 24]. Indeed, in this study, we observed that serum TNF-*α* and IL-1*β* levels in rats with UAN were significantly increased and significantly decreased, respectively, after XHYTF treatment. In addition, renal pathological sections revealed that inflammatory infiltration and interstitial fibrosis were significantly reduced. Masson staining results revealed that the proportion of collagen-positive area in the kidney was significantly reduced. AKT1, whose full name is RAC-*α* serine/threonine protein kinase, has been reported to play a role in the regulation of multiple biological effects of activated AKT, including regulation of cell proliferation, survival, and metabolism, and is one of the key molecules activated downstream of the PI3K signaling pathway [25]. It has been reported that the PI3K/AKT pathway can promote the occurrence of renal inflammation, autophagy, and fibrosis [26–28]. To further investigate whether the PI3K/AKT signaling pathway is involved in the mechanism of action of XHYTF in UAN, we detected the expressions of PI3K and AKT. The results revealed that XHYTF could reduce the expression of PI3K and AKT in rats with UAN, suggesting that XHYTF could regulate the PI3K/AKT signaling pathway while alleviating UAN. Therefore, the mechanism of action of XHYTF in treating UAN may be related to reducing the release of inflammatory factors and alleviating renal fibrosis; however, the specific molecular mechanism needs further study.

## 5. Conclusions

This study predicted, screened, analyzed, and verified potential active ingredients, core genes, and signaling pathways for XHYTF that may play a key role in biological processes through network pharmacology, molecular docking, and in vivo experiments. Our results indicated that XHYTF could alleviate UAN by targeting the PI3K/AKT signaling pathway, downregulating the expression of inflammatory cytokines, and reducing collagen fiber deposition. The mechanism of action of XHYTF in treating UAN might involve the synergistic effect of “multicomponents, multitargets, and multipathways.” Although these preliminary findings could not fully explain the potential mechanism of XHYTF in UAN, this study provided novel insights for further research. More studies are needed to explore the material basis and specific molecular mechanism of XHYTF in treating UAN, and more clinical studies are needed to verify our conclusions.

## Figures and Tables

**Table 1:** Basic information of TCM composition target of XHYTF.

Chinese pinyin name	Latin name	Number of components	Number of predicted targets
Yi-yi-ren	Coicis semen	6	28
Tu-fu-ling	Smilacis glabrae rhixoma	15	199
Tao-ren	Persicae semen	18	45
San-ci-gu	Pseudobulbus cremastrae seu pleiones	3	60
Ji-xue-teng	Spatholobus suberectus dunn	23	132
Huang-qi	Hedysarum multijugum maxim	19	239
Huang-bo	Phellodendri chinrnsis cortex	25	195
Di-long	Pheretima	34	175
Dang-shen	Codonopsis radix	17	104
Dan-shen	Radix salviae	60	130
Da-huang	Radix rhei et rhizome	10	71
Chuan-niu-xi	Cyathulae radix	4	171
Che-qian-zi	Plantaginis semen	7	152
Cang-zhu	Rhizoma atractylodis	4	58

## Data Availability

The data supporting the findings of this study will be made available from the corresponding author upon request.
